# To determine frequency of etiological factors in short statured patients presenting at an endocrine clinic of a tertiary care hospital

**Published:** 2014

**Authors:** Shazia Kulsom Lashari, Hussain Bux Korejo, Yasmeen Memon Memon

**Affiliations:** 1 Dr. Shazia Kulsom, MBBS, FCPS (Paeds), Senior Registrar, National Institute of Child Health (NICH), Karachi, Pakistan.; 2 Dr. Hussain Bux Korejo, MBBS, MCPS, FCPS (Paeds), Assistant Professor of Paediatrics, Liaquat University of Medical and Health Sciences, Jamshoro, Hyderabad, Sindh, Pakistan.; 3 Dr. Yasmeem Memon, FCPS Paeditrics, Associate Professor of Paediatrics, Liaquat University of Medical and Health Sciences, Jamshoro, Hyderabad, Sindh, Pakistan.

**Keywords:** Constitutional growth delay, Familial short stature, Growth hormone deficiency, Short stature

## Abstract

***Objective:*** To determine the frequency of etiological factors in short statured patients presenting at endocrine clinic of National Institute of Child Health, Karachi.

***Methods:*** This descriptive cross sectional study was conducted at Endocrine clinic of National Institute of Child Health, Karachi. One hundred children (48 boys and 52 girls) aged 3-15 years (mean 9.9±3.4) with short stature from January 2007 to July 2007 were evaluated during that period.

***Results:*** Constitutional growth delay (CGD) and familial short stature (FSS) **were** identified as the most common, 55% of all short stature cases. Non-endocrinal causes as a single entity was detected in 17 children. Most common etiological factors in order of frequency were normal variant of growth (CGD, FSS), Hypothyroidism, Growth Hormone deficiency (GHD), and Celiac disease. GHD was found in 13% of total cases and it comprises 44% among endocrinal causes. Boys outnumbered girls with ratio of 2.7:1 (p<0.05).

***Conclusion: ***Most common cause of short stature was normal variants of growth as a group. Children with height falling below 0.4^th^ percentile are more likely to have pathological cause.

## INTRODUCTION

Normal growth and development is a prime concern during childhood. Accurate assessment is essential for differentiating between normal and abnormal growth.^1^ In humans, growth is characterized by rapid height velocity during the first three years of life that decline progressively till the puberty growth spurt occurs.^2^ To define any growth point, one must measure children accurately and plot each point (height, weight, and head circumference) precisely. The short stature is categorized into three main types, primary growth abnormalities, secondary growth disorders and genetic short stature.^3^

The most common causes of short stature beyond the first year or two of life are familial short stature and constitutional growth delay.^4^^,^^5^ Almost any disease like renal, pulmonary and cardiac disease can cause growth failure. Celiac disease is a prime example of a remediable cause of short stature, especially in younger children.^6^ Therapies including glucocorticoids, chemotherapeutic drugs, surgery, radiotherapy and nutritional deprivation including decreased intake, malabsorption, increased resting energy expenditure or complications of restricted diet can result in growth failure.^7^ Common endocrine causes of growth failure and short stature are hypothyroidism,^8^^,^^9^ hypopitutarism (isolated GHD or multiple anterior pituitary hormone deficiencies),^10^^,^^11^ hypercortisolism (Cushing’s syndrome, exogenous and endogenous),^12^ and classical Laron syndrome^.,^ all are characterized by being overweight.^13^ Short stature is considered to be idiopathic if no causative disorder can be identified. Problem is being increasingly witnessed in Pakistan due to a wide range of factors.^14^ Among various causes, Growth Hormone Deficiency (GHD) even though considered uncommon is a treatable cause of short stature. Present study was aimed to see clinical profile of patients presenting with short stature at endocrine clinic of National Institute of Child Health, Karachi.

## METHODS

This descriptive cross sectional study was carried out by convenience sampling from January 2007 to July 2007 at endocrine clinic of National Institute of Child Health Karachi. The synopsis was approved by College of Physicians and Surgeons of Pakistan (CPSP), Karachi. In this period, 100 children with short stature were identified, by taking heights of children who came with complaints of short stature. Height was measured in children in centimeters, upper and lower segment ratio drawn by dividing total height by lower segment and plotted on CDC and NCHS growth charts. Only proportionate short stature patients were enrolled in study. Parents gave consent for the evaluation and inclusion in the study. The subjects included in the study were Children of both sex, age 3-15 year with significant short stature- 2SD who require evaluation with height inappropriate for parents. The exclusion criteria was short stature children with known syndrome (Noonan, Russelll Silver, Prader-willi) and patients with severe malnutrition were also excluded.

 History and physical examination was performed. Stage of puberty was determined according to the classification of Marshall and Tanner. Initial screening tests was done in all patients which included complete blood count, ESR, renal function test, Ca, P, Alk P,TSH, T4, T3, stool examination, urinalysis, and bone age radiographs. GHD was confirmed when peak GH concentration failed to reach 10ng/mL, with provocative test (Insulin tolerance test). Chromosomal study was done in females with short stature in whom other causes of short stature were excluded. Short stature was grouped as: (1) Normal variants of growth and (2) Pathologic short stature including non endocrine medical conditions, influencing growth and endocrine disorders. Normal variant group included CGD (i.e., proportionate short stature with a normal growth rate, delayed skeletal maturation often with a family history of delayed pubertal development, or late adolescent growth spurt) and FSS that is proportionate short stature with normal growth rate, skeletal age similar to chronologic age in the absence of any systemic disorders. The diagnosis of celiac disease was made by screening with anti tissue transglutaminase Ig A followed by histopathological examination of small gut biopsy. Data was analyzed by statistical package for social science (SPSS. 10).

## RESULTS

A total of 100 children with significant short stature -2SD were included in this study. Average age of children were 9.9 years, (±SD = ±3.4 years) with an age range of 3 – 15 years. Fifty two (52%) were female and 48 (48%) were male. Among 100 cases 73% had height below 0.4^th^ centile on NCHS growth charts. History of consanguity was present in 62 (62%) children (p-value < 0.0001). History of other short stature family member was present in 25 (25%) patients.

Three main etiological groups were identified. Normal variant of growth delay was the most common group (55%) in comparison to endocrinal diseases (28%) and non-endocrinal diseases (17%). In this study four most common single etiological factors were normal variant of growth delay (55%), hypothyroidism (15%), GHD (13%) and celiac disease (8%).

Among the endocrine causes out of 100 cases 13(13%) had GHD and 15 had hypothyroidism. Celiac disease was noticed as leading non-endocrinal cause of short stature in 8 patients. Other non-endocrine cause of short stature were chronic renal failure 3(3%), Turner Syndrome 3 (3%), familial rickets 2 (2%), chronic liver disease 1 (1%). All cases of GHD had age more then five year and 92.3% were falling below 0.4^th^ centile on NCHS growth chart.

**Table-I:** Common causes of short stature: No (%)

***Etiology ***	***Total***	***Male***	***Female***
Normal variant of growth	55 (55%)	29 (29%)	26 (26%)
Hypothyroidism	15 (15%)	3 (3%)	12 (12%)
Growth hormone deficiency	13 (13%)	9 (9%)	4 (4%)
Celiac disease	8 (8%)	4 (4%)	4 (4%)
Chronic renal failure	3 (3%)	2 (2%)	1 (1%)
Turners syndrome	3 (3%)	0 (0%)	3 (3%)
Familial rickets	2 (2%)	2 (2%)	0 (0%)
Chronic liver disease	1%	0%	1%

## DISCUSSION

In this study as single entity normal variants of growth (CGD and FSS) was responsible for 55% of short stature making it a very common cause. This observation is in accordance with worldwide studies^10^^,^^13^ and local study published by Sultan M et al. where CGD was observed in 37.9% making it commonest entity.^14^

Short stature is a frequent and important clinical presentation which pediatricians come across the worldover. As per definition 3% of the population falls in this category. Delay in diagnosis and initiation of treatment of an underlying disorder may result in failure to achieve the genetic potential in height. Fortunately most of the children with short stature are normal variant^3^, may be more than 65% of the short stature children.^15^ These normal variants of short stature need no medical treatment, only reassurance and growth monitoring is usually sufficient. On the other hand many serious and treatable diseases also cause and present with short stature alone or with other stigmata of that particular disease. These pathological processes need immediate recognition and timely treatment, to ensure normal height gain. Short stature has been studied very extensively worldwide, but such work is scanty in Pakistan.

Epidemiologic data indicates that all variants of normal growth are twice as common in boys as in girls. This gender difference may reflect greater concern about males who are shorter than their peers or who have delayed sexual development.^16^ In this study (28%) of short stature children were having endocrinal cause while non endocrinal causes contributed only 17%. Higher frequency of endocrinal cause especially GHD is present in almost all those studies conducted in referral/tertiary care hospitals. In comparison endocrinal contribution may be less than 05% in general population.^11^ In our study GHD contributed 13% almost twice as reported by Bhadda et al. (7.4%) in Indian population.^7^ GHD in Iran was reported 23.4% and 22.8% by Zargar.^13^ In this study GHD was more common in girls, which is also contrary to other studies. No precise reason for this difference was found. An other study from Multan which showed GHD in 18(10.7%) which is comparable with our results. He also concluded that normal variant of short stature as entity was more common than endocrine causes.^17^ Most common non endocrinal causes was celiac disease in 8(8%) out of total 100 cases that is consistent with many worldwide studies. In conclusion most common cause of short stature was normal variants of growth as a group. In males it was CGD and in females FSS. Same conclusion was reported in India.^18^ Another important observation made in this study is that all cases of pathological short stature were having marked growth retardation, height falling below 0.4^th^ percentile on NCHS growth charts as compared to normal variants, where only 40% cases were falling below 0.4^th^ percentile. Limitations of this study include short follow up which is not as desired for such studies. 

**Table-II:** Comparison of etiology of short stature with other studies

	***Lindsey R et al.*** ^19^ *** (%)*** ***n=555***	***Moayeri H et al.*** ^20 ^ ***(%)*** ***n=426***	***Bhadda SK et al.*** ^7^ *** (%)*** ***n=352***	***Colaco P et al.*** ^6 ^ ***(%)*** ***n=200***	***Own study (%)*** ***n=100***
Normal variant of growth	80%	47%	15.9%	20.5%	30.1%
GHD	2.5%	23.4%	7.4%	19.5%	13%
Turner Syndrome	1.5%	4.5%	7.4%	7.4%	3%
Hypothyroidism	01%	08%	14.2%	10%	15%
Chronic systemic diseases	10%	04%	12.4%	8.5%	17%

## CONCLUSION

Most children with short stature do not have an endocrine disorder. Children with height falling below 0.4^th^ percentile are more likely to have pathological cause. This study suggests the presence of significant difference in etiology of short stature in our population compared to other parts of the world.

## Recommendations:

1. Growth hormone analysis is not needed in every child with short stature. Growth velocity monitoring is very sensitive measure, which can be used, in almost half cases of short stature for evaluation without taking much risk.
2. Screening children for short stature should be done if the height is falling below 0.4^th^ percentile when plotted on NCHS growth charts.
3. Further multi-centered studies are required from different parts of the country to assess the above-mentioned etiological profile of short stature in Pakistan.

## Author's contribution:

**SK: **Conceived, designed and statistical analysis of data.

**HBK: **Data collection and manuscript writing.

**YM:** Did final review and approval of manuscript.
